# Regulation of carbohydrate degradation pathways in *Pseudomonas* involves a versatile set of transcriptional regulators

**DOI:** 10.1111/1751-7915.13263

**Published:** 2018-04-02

**Authors:** Zulema Udaondo, Juan‐Luis Ramos, Ana Segura, Tino Krell, Abdelali Daddaoua

**Affiliations:** ^1^ Department of Biomedical Informatics University of Arkansas for Medical Sciences 4301W. Markham St., Slot 782 Little Rock AR 72205 USA; ^2^ Department of Environmental Protection Estación Experimental del Zaidín C/ Profesor Albareda 1 E‐18008 Granada Spain; ^3^ Department of Biochemistry and Molecular Biology II Pharmacy School Granada University Granada Spain

## Abstract

Bacteria of the genus *Pseudomonas* are widespread in nature. In the last decades, members of this genus, especially *Pseudomonas aeruginosa* and *Pseudomonas putida*, have acquired great interest because of their interactions with higher organisms. *Pseudomonas aeruginosa* is an opportunistic pathogen that colonizes the lung of cystic fibrosis patients, while *P. putida* is a soil bacterium able to establish a positive interaction with the plant rhizosphere. Members of *Pseudomonas* genus have a robust metabolism for amino acids and organic acids as well as aromatic compounds; however, these microbes metabolize a very limited number of sugars. Interestingly, they have three‐pronged metabolic system to generate 6‐phosphogluconate from glucose suggesting an adaptation to efficiently consume this sugar. This review focuses on the description of the regulatory network of glucose utilization in *Pseudomonas*, highlighting the differences between *P. putida* and *P. aeruginosa*. Most interestingly, It is highlighted a functional link between glucose assimilation and exotoxin A production in *P. aeruginosa*. The physiological relevance of this connection remains unclear, and it needs to be established whether a similar relationship is also found in other bacteria.

## Introduction

Bacteria of the genus *Pseudomonas* are ubiquitous inhabitants of soil, water, plant surfaces, animal and human tissue and have a robust metabolism for amino acids and organic acids as well as aromatic compounds (Jiménez *et al*., 2002; Puchalka *et al*., 2008; Valerie *et al*., 2013; Daniels *et al*., 2010), and the deciphering of the complete genomes of a number of *Pseudomonas* strains from different species has revealed that these microbes metabolize a very limited number of sugars (Buell *et al*., 2003; Feil *et al*., 2005; Joardar *et al*., 2005), which are mainly glucose, glucuronic acid and fructose (Daniels *et al*., 2010). This metabolic pattern has been associated with their lifestyle as they inhabit environmental niches characterized by a limited presence of sugars (Silby *et al*., 2011).

**Figure 1:**
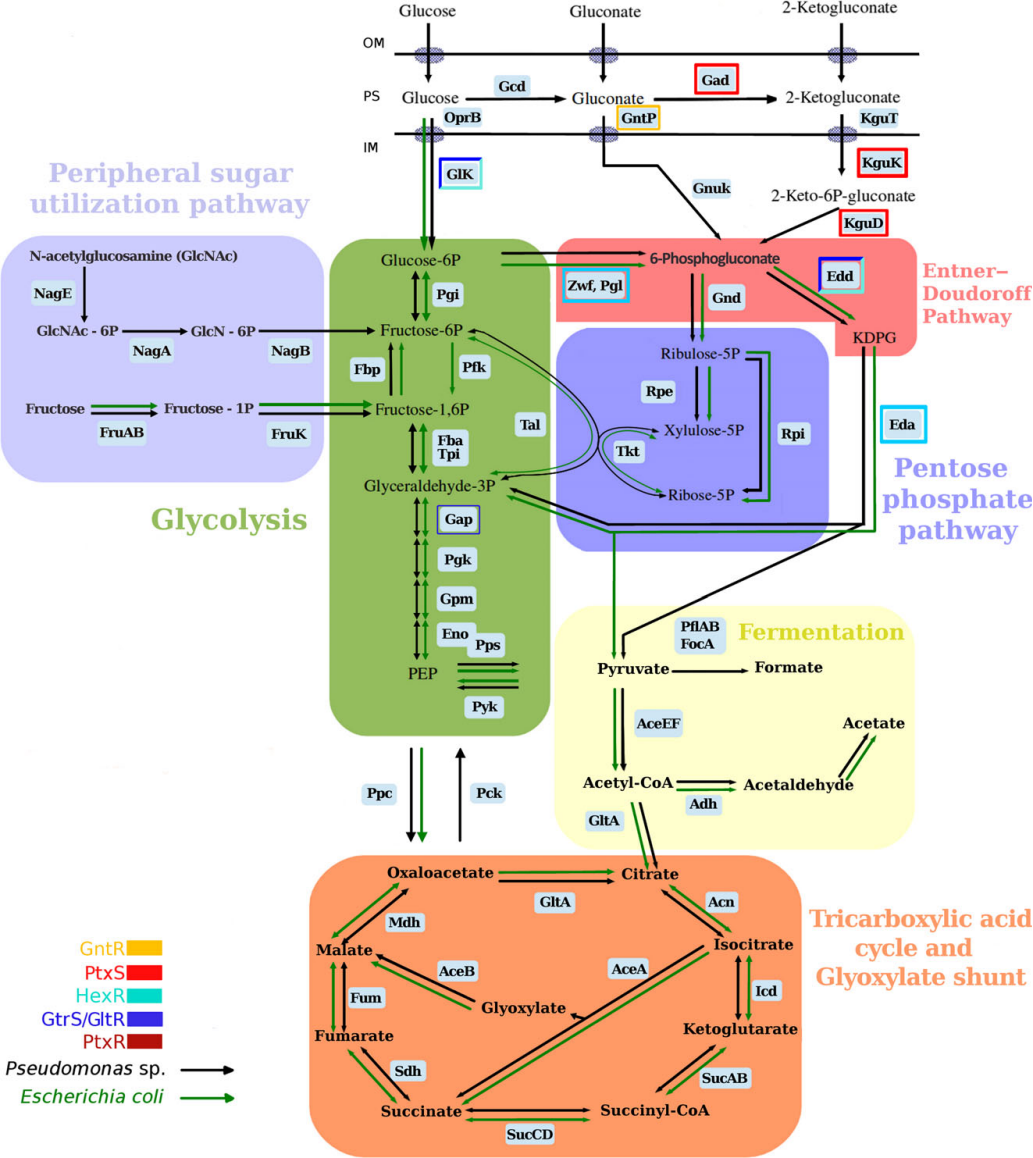
Schematic representation of the glucose metabolism in *Pseudomonas* and *Escherichia coli* as deduced from gene annotations and functional analysis in the wild‐type strain. Genes whose expression is controlled by the regulators described in this review are boxed in different colours.

Studies in *Pseudomonas putida* have shown that there is a three‐pronged metabolic system to generate 6‐phosphogluconate from glucose (Del Castillo *et al*., 2007; Fig. 1). Glucose enters through the OprB porin into the periplasmic space. Once in the periplasm, glucose can be either transported to the cytosol or converted into gluconate and 2‐ketogluconate (KG) to be subsequently transferred to the cytosol via different transporters (GntP and KguT respectively). Gluconate can be phosphorylated to 6‐phosphogluconate by gluconokinase (GnuK), whereas the conversion of 2‐ketogluconate into 6‐phosphogluconate requires two enzymatic reactions mediated by KguK and KguD (Fig. 1); whereby, it starts the Entner–Doudoroff pathway (Nikel *et al*., 2015).

**Figure 2:**
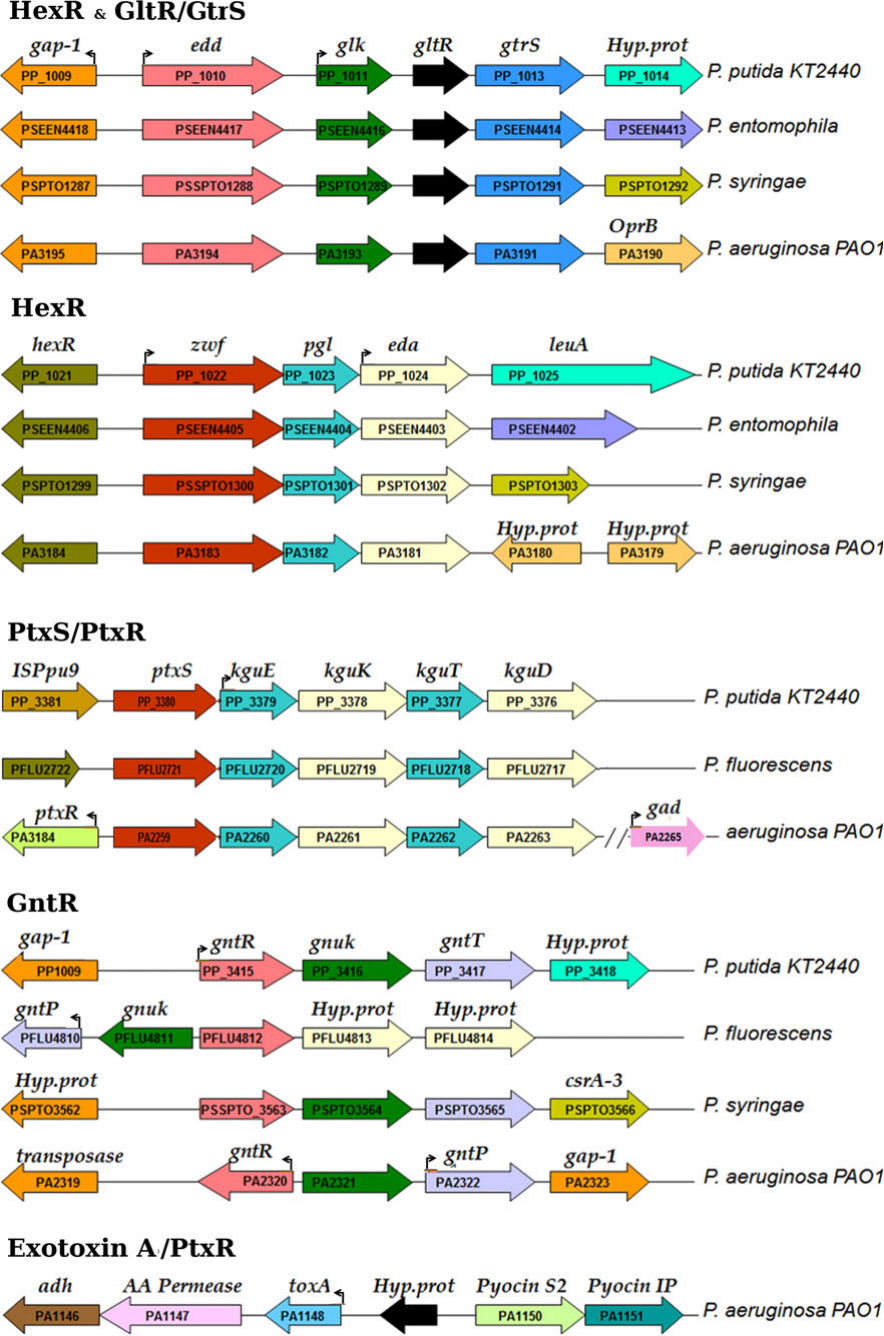
Genetic organization of genes encoding enzymes of carbohydrate degradation pathways and exotoxin A in different *Pseudomonas* strains. The genes that were found to be regulated are boxed, and the corresponding regulator y system is provided over each block of genes.

**Figure 3:**
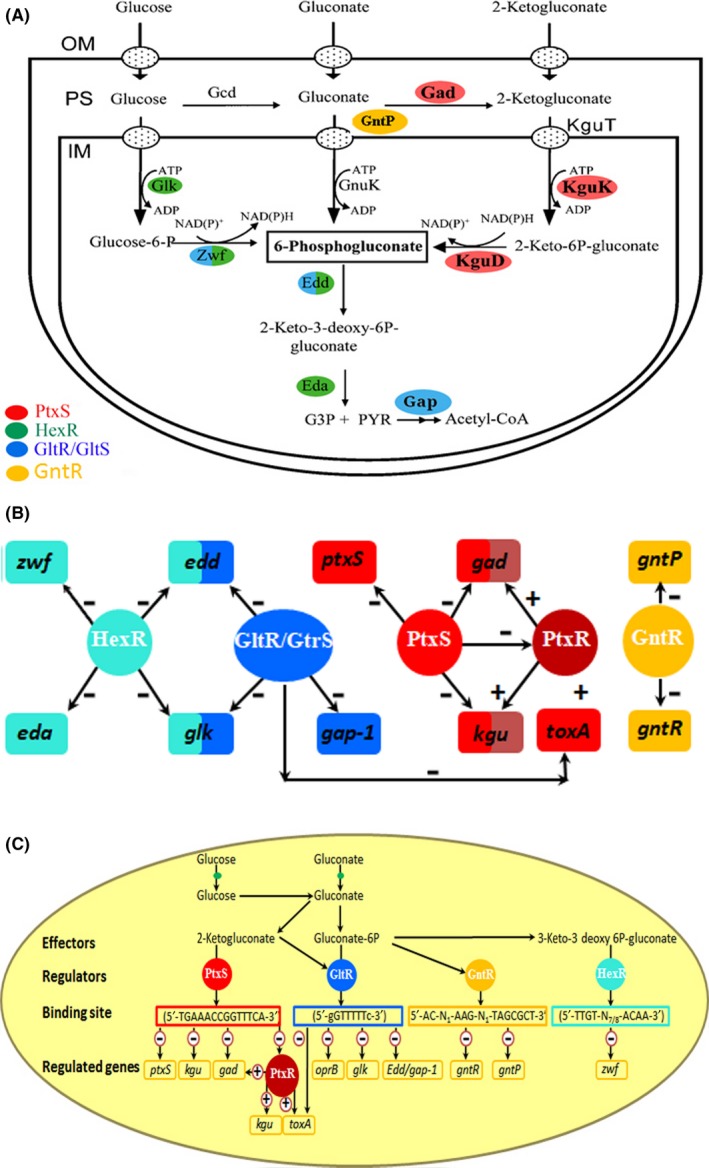
Schematic view of the concerted regulation of gene expression involved in glucose metabolism. A. Schematic view of the three internalization routes. B. The functional interconnectivity of the five regulatory mechanisms. C. Summary of information flow from effectors to regulated genes. The − and + symbols indicate repression or activation of gene expression respectively.

In addition, the growing number of complete genome sequences of *Pseudomonas* strains in public databases (Jayal *et al*., 2017; Nesme *et al*., 2017; Wilson *et al*., 2017) along with the increasing sophistication of the techniques used in metabolomics and transcriptomics (La Rosa *et al*., 2015; Nikel *et al*., 2015) has provided us with key information for a further understanding of the complex regulation processes and functionality of the enzymes that participate in carbohydrate metabolism in *Pseudomonas*. The genes encoding the carbohydrate catabolic pathways are organized in operons (Fig. 2), which are under the control of different regulators that respond differentially to distinct pathway intermediates, suggesting a hierarchy in the control of glucose metabolism related to a tight gene expression (Rojo, 2010; Daddaoua *et al*., 2014; Figs 2 and 3).

Transcriptional regulation is the primary mechanism to control gene expression in prokaryotic cells (Ishihama, 2000). Typically, transcriptional regulators sense certain environmental cues and the resulting molecular stimulus modulates their interactions with RNA polymerases or DNA. In one‐component regulatory system (OCS), the input (i.e. sensing) and output functions are united in a single protein. In two‐component system (TCS), a membrane‐bound histidine kinase is dedicated to signal sensing, whereas the response regulator protein mediates a transcriptional response (Mitrophanov and Groisman, 2008). TCS represents the major regulatory mechanism in bacteria and archaea and is responsible for the transformation of external and internal stimuli into adaptive responses, including regulation of gene expression and methylation of target proteins (Skerker *et al*., 2005; Mitrophanov and Groisman, 2008). In a typical ligand‐induced TCS, changes in the autokinase activity of the sensor kinase modulate the rate of transphosphorylation to its cognate response regulator, which in turn defines the system output. In addition, there is evidence for TCS that contains additional signalling proteins (Mitchell *et al*., 2015; Popella *et al*., 2016).

Data so far available indicate that the regulation of the glucose catabolic pathways in *Pseudomonas is* controlled by the concerted action of the one‐component systems (OCSs) HexR, PtxS, PtxR and GntR as well as the two‐component system (TCS) GltR/GtrS (Daddaoua *et al*., 2009, 2012, 2014, 2017). This review focuses on the description of this regulatory network and highlights a number of differences in regulation between strains of the soil bacterium *P. putida* and the opportunistic human pathogen *Pseudomonas aeruginosa*. Glucose metabolism in Pseudomonads is fundamentally different to that in *Escherichia coli* (Lendenmann *et al*., 1996; Fuhrer *et al*., 2005; Fig. 1), and the current knowledge on its regulation is reviewed here.

## Carbohydrate catabolic pathways in *Pseudomonas*

Metabolic flux analysis of wild‐type *Pseudomonas* and different mutant strains has permitted to estimate the carbon flow through the individual three peripheral uptake routes. These data revealed that the flow through the gluconate kinase (GnuK) route is minor, whereas the remaining flux appears to be similarly distributed to the routes that involve the phosphorylation of either glucose or 2‐ketogluconate (Del Castillo *et al*., 2007). Carbohydrates enter the periplasmic space through porins located in the outer membrane (OprB1/OprB2) (Figs 1 and 3A). Once in the periplasm, glucose can be oxidized to gluconate through the action of a glucose dehydrogenase (Gcd), and gluconate is transported to the cytoplasm and phosphorylated to 6‐phosphogluconate (6PG) by gluconate kinase (GnuK). Alternatively, gluconate, still in the periplasm, can be further oxidized to 2‐ketogluconate (2KG) by the action of gluconate dehydrogenase (Gad); then, 2KG enters the cytoplasm and is converted into 6PG via 2‐keto‐6‐phosphogluconate by the action of the 2‐ketogluconate kinase (KguK) and 2‐ketogluconate‐6‐phosphate reductase (KguD). Glucose can also be transported directly to the cytoplasm through an ABC uptake system, and the first acting enzyme is glucokinase (Glk) that phosphorylates glucose to give glucose 6‐phosphate (G6P). Next, the combined action of glucose 6‐phosphate dehydrogenase (Zwf) and 6‐phosphogluconolactonase (Pgl) convert G6P into 6‐phosphogluconate (6PG) (Del Castillo *et al*., 2007). The produced 6PG enters to the Entner–Doudoroff route and is converted into 2‐keto‐3‐deoxy‐6‐phosphogluconate (KDPG) by the action of the 6‐phosphogluconate dehydratase (Edd) and then hydrolysed to produce glyceraldehyde‐3‐phosphate and pyruvate by the action of the 2‐keto‐3‐deoxy‐6‐phosphogluconate aldolase (Eda) (Figs 1 and 3A; Braga *et al*., 2004). Glyceraldehyde‐3‐phosphate is further metabolized by the glyceraldehyde‐3‐phosphate dehydrogenase (Gap‐1) to d‐glycerate 1,3‐bisphosphate, while pyruvate is decarboxylated to acetyl‐coenzyme A (Acetyl‐CoA) and enters the Krebs cycle (Fig. 1).

## Genomic distribution of genes involved in *Pseudomonas* carbohydrate catabolism

The analysis of the genomic localization of genes involved in glucose catabolism (Fig. 2) revealed that genes are arranged in operons that encode for different sets of catabolic enzymes, some transcriptional regulators as well as specific porins. Interestingly, the two genes that encode the key enzymes of the Entner–Doudoroff pathway, *edd* and *eda*, are located in different operons. The *edd* and *glk* genes (phosphorylative branch) form part of the same operon together with the genes encoding the regulatory proteins GltR, GtrS and the *gap‐1* gene (Fig. 2). The *eda* gene forms an operon with the *zwf* and *pgl* genes (phosphorylative branch) as well as the gene encoding the regulatory protein HexR (Fig. 2). This genomic organization (*edd*/*glk* and *zwf*/*pgl*/*eda* containing operons) suggests a complex regulation, because the main route of glucose metabolism in *Pseudomonads* occurs through the 2KG degradation pathway, which, however, are encoded by *kguT, kguK* and *kguD* that form another operon with *ptxS*, encoding a transcriptional regulator (Fig. 2). The fact that the genes that control the expression of Edd and Eda proteins are located in the same operons as the genes involved in the glucose phosphorylative pathway, which is not the main glucose degradative pathway in *Pseudomonas,* can be a reminiscence of an ancestral organism and explains why this pathway is still active in *Pseudomonas*; both *edd* and *eda* genes are necessary for the 6PG conversion into tricarboxylic acid (TCA) intermediates, regardless of the peripheral pathway by which glucose has been converted into 6PG.

## Transcriptional regulators involved in *Pseudomonas* carbohydrate degradation pathway

In *E. coli*, 300 types of transcription factors have been identified and about 10% of them are TCSs (Yamamoto *et al*., 2005; Ishihama *et al*., 2014). In bacteria, extracellular signals are recognized predominantly by TCSs (Hoch, 2000; Stock *et al*., 2000; Mascher, 2006; Gao *et al*., 2007; Lacal *et al*., 2010) which typically recognize signals at extracytoplasmic ligand binding domains.

Usually, promoters that control the expression of genes that encode proteins implicated in the synthesis of cell structures, such as flagella, pili and fimbriae, or those involved in complex cellular processes, that is, virulence or biofilm formation, are often controlled by multiple environmental signals that are recognized by multiple transcription factors (Dalebroux *et al*., 2010; Rasamiravaka *et al*., 2015; ).

Data currently available indicate that the control of glucose metabolism in *Pseudomonas* is controlled by the concerted action of the HexR, PtxS, PtxR and GntR, which are OCSs, whereas and the GltR/GtrS is TCS. The transcriptional regulation of glucose degradation has been studied in *Pseudomonas putida* KT2440 and *P. aeruginosa* PAO1, and the current knowledge is summarized in Figure 1.

### OCS involved in carbohydrate catabolism pathways

#### HexR

The HexR regulator belongs to the RpiR family of transcriptional regulators whose members typically act as transcriptional regulators in sugar catabolism and have been identified as both repressors and activators in Gram‐negative and Gram‐positive bacteria (Yamamoto *et al*., 2001). In *E. coli*, RpiR negatively regulates the expression of the *rpiB* gene that encodes a ribose 5‐phosphate isomerase, an enzyme that catalyses the reversible reaction of ribose 5‐phosphate to ribulose 5‐phosphate and forms part of the pentose phosphate pathway (Fig. 1; Sorensen and Hove‐Jensen, 1996). Sugar‐responsive RpiR proteins form dimers in solution and have an N‐terminal helix–turn–helix (HTH) DNA‐binding motif and a sugar isomerase‐like binding (SIS) domain at their C‐terminal extension (Bateman, 1999).

**Figure 4:**
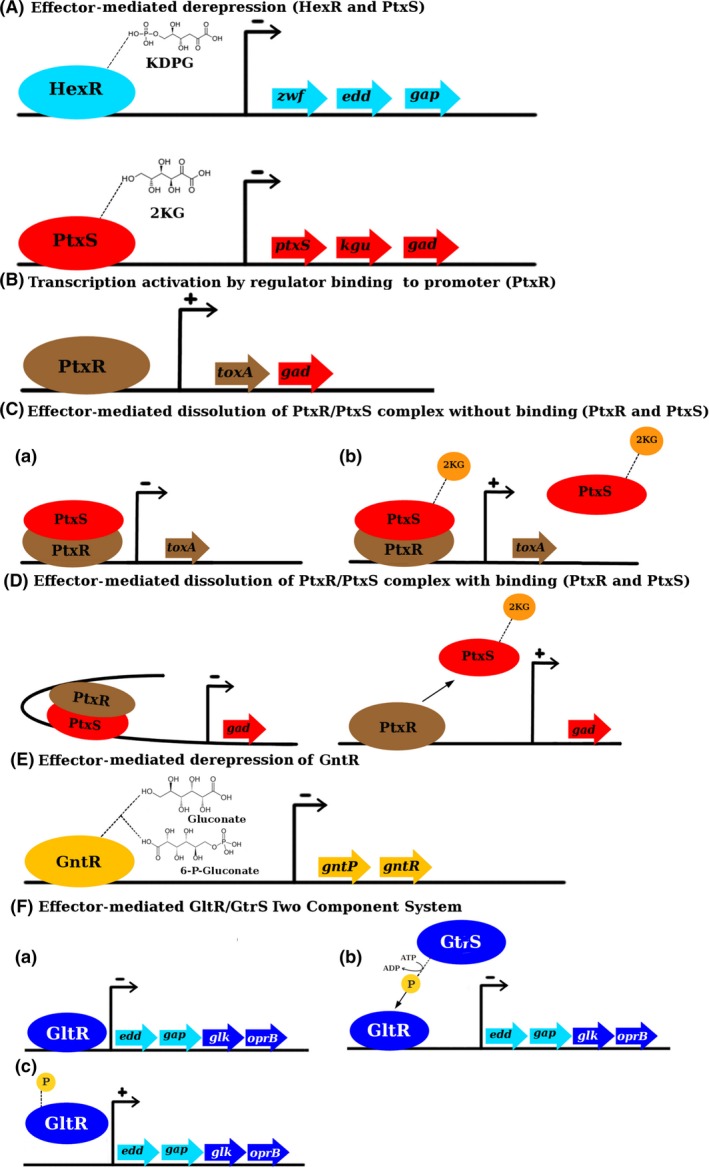
The mechanisms’ action of transcriptional regulators involved in the regulation of carbohydrate catabolism pathways. The − and + symbols indicate repression or activation of gene expression respectively. A. Effector‐mediated derepression (HexR and PtxS). B. Transcription activation by regulator binding to promotor (PtxR). C. Effector‐mediated dissolution PtxR/PtxS complex without binding (PtxR and PtxS). D. Effector‐mediated dissolution of PtxR/PtxS complex with binding (PtxR and PtxS). E. Effector‐mediated derepression of GntR. F. Effector‐mediated GltR/GtrS two component system.

In *Pseudomonas*, HexR regulator is divergently transcribed from the *zwf/pgl/eda* operon, and the physical organization of these genes is highly conserved within *Pseudomonas*, which suggests the conservation of the identified regulatory mechanism (Fig. 2). HexR regulator controls the *zwf/pgl/eda* and *edd/glk/gltR‐2* operons as well as the *gap‐1* gene (Daddaoua *et al*., 2009; Fig. 3 and Table 2) and is also required for the metabolism of other sugars such as fructose and gluconate (Fig. 1). The HexR regulator exerts its regulatory action by binding to specific sequences in the target promoters (Figs 3 and 4 and Table 1), which in turn prevents the progress of RNA polymerase. HexR recognizes 2‐keto‐3‐deoxy‐6‐phosphogluconate (KDPG), an intermediate of the Entner–Doudoroff pathway (Table 2), and is required not only for glucose catabolism but also for the metabolism of other sugars such as fructose and gluconate (Fig. 1). HexR acts as a transcriptional repressor in the absence of a specific effector; but binding of KDPG to DNA‐bound HexR causes protein dissociation and transcriptional activation (Fig. 4). Furthermore, it has been speculated that this is not a ‘random genetic organization’ as KDPG plays a relevant role as a signalling molecule in catabolite repression and in the response to oxidative stress in *Pseudomonas* (Daddaoua *et al*., 2009; Rojo, 2010).

**Table 1:** Regulatory proteins involved in the transcriptional control of *Pseudomonas* carbohydrate catabolic pathways

Regulator	Activation of genes	Repression of genes	Effector	Reference
HexR	None	*edd, gap‐1, zwf*	KDPG	Daddaoua *et al*. (2009) and Del Castillo *et al*. (2007)
PtxS	None	*ptxS, kgu, gad, ptxR, toxA*	2‐ketogluconate	Hamood *et al*. (1996) and Daddaoua *et al*. (2010, 2012)
PtxR	*toxA*	None	Unknown	Hammod *et al*. (1996) and Daddaoua *et al*. (2012, 2013)
GltR/GtrS	*edd, gap‐1*,* glk*,* oprB, toxA*	None	6‐phosphogluconate and 2‐ketogluconate	Daddaoua *et al*. (2014)
GntR	None	*gntR, gntP*	Gluconate and 6‐phosphogluconate	Daddaoua *et al*. (2017), Izu *et al*. (1997), and Peekhaus and Conway (1998)

**Table 2:** Specific regulator binding site involved in *Pseudomonas* carbohydrate catabolic pathways

Promoter	Regulator	Effector	Sequence of operator site	Position of operator	*K*_D_	Reference
*zwf*	HexR	KDPG	5′‐TTGT‐N7/8‐ACAA‐3′	+30; +1	780 ± 40 nM	Daddaoua *et al*. (2009)
*edd*	HexR	KDPG	5′‐TTGT‐N7/8‐ACAA‐3′	+16; +41	774 ± 80 nM	Daddaoua *et al*. (2009)
*gap‐1*	HexR	KDPG	5′‐TTGT‐N7/8‐ACAA‐3′	−6; −18	480 ± 50 nM	Daddaoua *et al*. (2009)
*ptxS*	PtxS	2 ketogluconate	5′‐TGAAACCGGTTTCA‐3′	+5; −9	ND	Daddaoua *et al*. (2010, 2012)
*kgu*	PtxS	2 ketogluconate	5′‐TGAAACCGGTTTCA‐3′	+5; +18	ND	Daddaoua *et al*. (2010, 2012)
*gad*	PtxS	2 ketogluconate	5′‐TGAAACCGGTTTCA‐3′	+10; +23	ND	Daddaoua *et al*. (2010, 2012)
*toxA*	PtxS	2 ketogluconate	5′‐TGAAACCGGTTTCA‐3′	ND	ND	Daddaoua *et al*. (2010, 2012)
*gad*	PtxR	Unknown	5′‐CGGCGCGCCCG‐3′	−32; −41	ND	Daddaoua *et al*. (2010, 2012, 2013)
*kgu*	PtxR	Unknown	5′‐CGGCGCGCCCG‐3′	−34; −43	ND	Daddaoua *et al*. (2010, 2012, 2013)
*toxA*	PtxR	Unknown	5′‐CGGCGCGCCCG‐3′	−42; −51	ND	Daddaoua *et al*. (2010, 2012, 2013)
*oprB*	GltR/GtrS	2‐ketogluconate and Gluconate	5′‐gGTTTTTc ‐3′	−10; −21	ND	Daddaoua *et al*. (2014)
*glk*	GltR/GtrS	2‐ketogluconate and Gluconate	5′‐gGTTTTTc ‐3′	+22; +33	ND	Daddaoua *et al*. (2014)
*edd*	GltR/GtrS	2‐ketogluconate and Gluconate	5′‐gGTTTTTc ‐3′	−4; +9	ND	Daddaoua *et al*. (2014)
*gap‐1*	GltR/GtrS	2‐ketogluconate and Gluconate	5′‐gGTTTTTc ‐3′	−12; +2	ND	Daddaoua *et al*. (2014)
*toxA*	GltR/GtrS	2‐ketogluconate and Gluconate	5′‐gGTTTTTc ‐3′	+177; +190	ND	Daddaoua *et al*. (2014)
*gntR*	GntR	Gluconate and 6‐phosphogluconate	5′‐AC‐N_1_‐AAG‐N_1_‐TAGCGCT‐3′	−4, −17	≈1 mM	Daddaoua *et al*. (2017)
*gntP*	GntR	Gluconate and 6‐phosphogluconate	5′‐AC‐N_1_‐AAG‐N_1_‐TAGCGCT‐3′	−4, −17	≈1 mM	Daddaoua *et al*. (2017)

**Figure 5:**
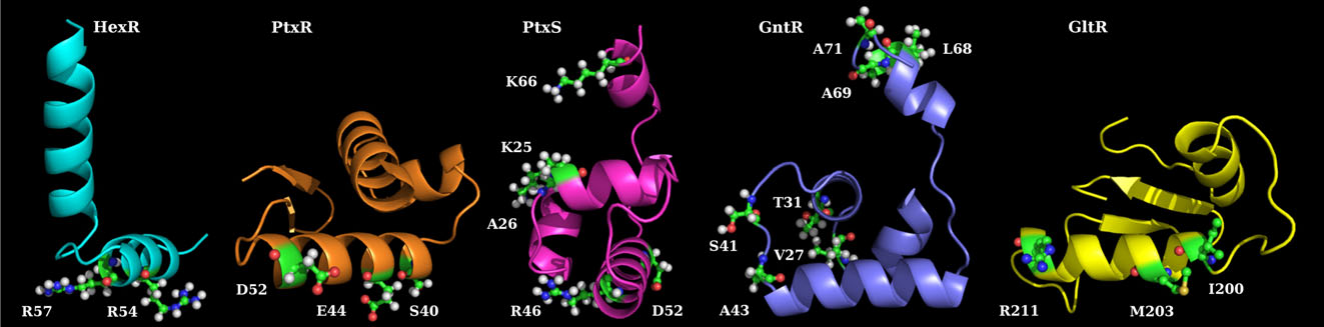
Homology models of NH_2_ or COOH‐terminal extensions corresponding to the DNA‐binding domains of regulators involved in carbohydrate catabolism pathways. The amino acids involved in the interaction of the regulator with DNA are highlighted.

Homology modelling of HexR using the structure of the YbbH transcriptional regulator from *Bacillus subtilis* (PDB: 2O3F) as a template (36% sequence identity) revealed that HexR is composed of two distinct domains, namely an N‐terminal HTH containing DNA‐binding domain (residues 20‐126) and a C‐terminal domain (residues 117‐256) that is homologous to the phosphosugar isomerase domain of the RpiR family. Furthermore, the inspection of this model suggested that the amino acids involved in DNA recognition are Gln‐43, Lys‐46, Glu‐49, Arg‐54 and Arg‐57, and the amino acids Arg‐54 and Arg‐57 may be directly interacting with DNA (Fig. 5; Daddaoua *et al*., 2009).

Finally, the HexR operator sites have been identified in the *zwf*,* edd* and *gap‐1* promoters of *P. putida* KT2440, what permitted the definition of the consensus sequence 5′‐TTGT‐N_7/8_‐ACAA‐3′, which likely corresponds to the specific HexR binding motif (Daddaoua *et al*., 2009; Table 2 and Fig. 3).

#### PtxS and PtxR

In *Pseudomonas*, the *ptxS* gene is located next to the *kguE/kguK/kguT/*and/*kguD genes* (Fig. 2). Similarly, to other members of the well‐characterized LacI family, PtxS has an N‐terminal DNA‐binding domain and a C‐terminal effector binding domain. In several species of the genus *Pseudomonas*, the role of PtxS in the control of the gluconate degradation pathway has been elucidated (Daddaoua *et al*., 2012; Suresh *et al*., 2014). In *P. aeruginosa* PAO1, PtxS binds to the palindromic sequence 5′‐TGAAACCGGTTTCA‐3′ (Fig. 3 and Table 2) located near the ‐10 region repressing the expression of the corresponding gene (Table 1; Daddaoua *et al*., 2010). The three‐dimensional model of the PtxS N‐terminal domain (PF00356) was generated with I‐TASSER server (C‐score: 0.74) based on the transcriptional regulator CcpA (Schumacher *et al*., 2006, 2011; PDB: 1ZVV and 3OQMA) that shares 26% sequence identity with PtxS. The model suggested that amino acids Lys‐25, Ala‐26, Arg‐46, Asp‐52 and Lys‐66 are important for DNA recognition (Yang *et al*., 2015; Fig. 5).

In *P. putida*, KT2440 PtxS controls its own expression as well as that of the operons *gad* and *kgu* (Daddaoua *et al*., 2012). However, in *P. aeruginosa* PAO1, PtxS regulates, in addition (Figs 1 and 3), the expression of the *toxA* gene that encodes the exotoxin A, a primary virulence factor (Daddaoua *et al*., 2012; Fig. 3). This protein is an ADP‐ribosyl transferase that irreversibly inhibits protein synthesis in eukaryotic cells causing cell death.

In *P. aeruginosa*, but not in other *Pseudomonas,* an additional transcriptional regulator *ptxR* is located within the *kgu* cluster and transcribed divergently from the *ptxS* gene (Fig. 2). PtxR belongs to the LysR‐type of transcriptional regulators and does not share any significant sequence similarities with PtxS (13% sequence identity in an alignment with seven gaps). PtxR is also involved in the regulation of *toxA* expression (Hamood *et al*., 1996) which encodes an ADP‐ribosyl transferase that irreversibly inhibits protein synthesis in eukaryotic cells causing cell death. It has been shown that endotoxin A production is triggered by certain environmental conditions (such as cation concentration, iron and oxygen levels or temperature) and is controlled by different regulators, and the involvement of RegA, PtxR and the iron‐starvation alternative sigma factor PvdS in this complex regulatory process has been documented (Wick *et al*., 1990; Hamood *et al*., 2004).

PtxR is also involved in the regulation of several genes of the glucose degradation pathway (Daddaoua *et al*., 2012) and recognizes a pseudopalindrome with a consensus sequence of 5′‐ GGC‐N_4‐6_‐GCC ‐3′ (Fig. 3 and Table 2) which overlapped with the RNA polymerase binding site of P_*toxA*_, P_*kgu*_ and P_*gad*_ promoters. PtxR has a DNA‐binding HTH domain (in the N‐terminal region) and has a signal receptor domain at its C‐terminal extension that is composed of two subdomains: one is responsible for inducer recognition, whereas the other is involved in the response (Maddocks and Oyston, 2008). A three‐dimensional homology model of PtxR was built using, the LysR family protein member, CrgA of *Neisseria meningitidis* (PDB: 3 hhg) (Sainsbury *et al*., 2009) as a template. The *in silico* analysis of the model together with isothermal titration calorimetry (ITC) assays using PtxR mutants revealed that residues Ser‐40, Glu‐44 and Asp‐52 in the HTH are involved in interaction with the target DNA (Fig. 5).

In *P. aeruginosa*, PtxS regulates the expression of *ptxR*; therefore, it is involved in the indirect control of the production of exotoxin A (Fig. 3). PtxS does not directly bind to the *toxA* promoter; instead, ITC analyses demonstrated that PtxS forms a tight complex with PtxR, either in its free form or when bound to the P_*toxA*_ DNA (Colmer and Hamood, 1998; Daddaoua *et al*., 2012; ). The binding of PtxS to DNA‐bound PtxR prevents PtxR from activating transcription (Fig. 4). The binding of 2‐ketogluconate to PtxS causes its dissociation from PtxR allowing the activation of transcription (Daddaoua *et al*., 2012). It could be speculated that PtxR might be responsible for the recruitment of RNA polymerase allowing transcription.

In contrast to the mechanism by which PtxR and PtxS control the expression of P_*toxA*_ *,* both regulators bind to P_*kgu*_ and P_*gad*_. As both proteins interact with each other and as their operator sites are separated by around 50 bp, it has been shown that the interaction between these two DNA‐bound provokes the formation of a DNA loop (Daddaoua *et al*., 2013). It has been suggested that this loop structure prevents the RNA polymerase to access the promoter (Huo *et al*., 2009).

The binding of 2‐ketogluconate to PtxS breaks the loop permitting RNA polymerase recruitment for the transcription of the genes involved in 2‐ketogluconate catabolism (Fig. 4; Daddaoua *et al*., 2012, 2013). Therefore, it seems that there are two different control mechanisms exerted by the same regulator, in the P_*toxA*_ promoter via PtxS/PtxR/DNA complex formation and in the case of P_*kgu*_ and P_*gad*_ promoters by forming a DNA/PtxS/PtxR/DNA loop structure (Fig. 4).

#### GntR

As has been mentioned by Daddaoua *et al*. (2017), the inspection of the genetic context of genes involved in glucose metabolism in *P. aeruginosa* resulted in the detection of a GntR‐like transcriptional regulator (Jain, 2015), that is predicted to possess an N‐terminal HTH DNA‐binding motif and a periplasmic binding protein‐like domain for effector binding (Daddaoua *et al*., 2017). In *P. aeruginosa*, it was found that the *gntR* gene is transcribed divergently to the *gnuK* gene (gluconokinase), which is located adjacent to those of the gluconate transporter (GntP) and a glyceraldehyde‐3‐phosphate dehydrogenase (GapN) (Fig. 2) It has been proposed that GntR regulates its own expression, as well as that of a gluconokinase (GnuK), gluconate permease (GntP) and gluconate 6‐phosphate dehydrogenase (GntZ) gene (Daddaoua *et al*., 2017; Del Castillo *et al*., 2007).

However, the GntR homologue of *P. aeruginosa* shared only modest sequence identities (11–37%) with characterized paralogues in *Corynebacterium glutamicum* (Frunzke *et al*., 2008), *Sinorhizobium meliloti* (Steele *et al*., 2009) and *Vibrio cholerae* (Roy *et al*., 2016). Recent data confirm that GntR represses its own expression as well as that of the GntP gluconate permease (Table 1). In contrast to PtxS and GtrS/GltR, GntR did not modulate expression of the *toxA* gene encoding the *P. aeruginosa* exotoxin A virulence factor. GntR bound to promoters P_*gntR*_ and P_*gntP*_, and the consensus sequence of its operator was defined as 5′‐AC‐N_1_‐AAG‐N_1_‐TAGCGCT‐3′ (Table 2). Both operator sites overlapped with the RNA polymerase binding site. GntR employs an effector‐mediated derepression mechanism (Fig. 4) because the release of promoter‐bound GntR is induced by gluconate and 6‐phosphogluconate that bind with similar apparent affinities to the GntR/DNA complex (Table 2). Surprisingly, GntR and PtxS are paralogous which may have evolved from a common ancestor (Daddaoua *et al*., 2017).

The three‐dimensional model of the GntR N‐terminal domain was generated with the I‐TASSER server (C‐score: 0.48) based on the transcriptional regulator from *Bacillus subtilis* (PDB: 1ZVV) which had 25% of identity with GntR (Schumacher *et al*., 2006). The analysis of the model suggested that the amino acids Val‐27, Tyr‐31, Ser‐41, Ala‐43, Leu‐68, Ala‐69 and Ala‐71 were important for the recognition of its target DNA (Fig. 5).

### Role of the GltR/GtrS (TCS) in the regulation of the carbohydrate catabolism

The TCS GtrS/GltR was also found to participate in the transcriptional regulation of glucose catabolism. Different research groups have provided initial information on the role GtrS and GltR, but they did not release that they do form a TCS. Whereas GltR was identified to be essential for efficient glucose transport (Sage *et al*., 1996), GtrS was found to be important for optimal host colonization and dissemination in a mouse infection model by modulating type III secretion in response to host cells (O'Callaghan *et al*., 2012). However, Daddaoua *et al*. (2014) demonstrated that the GltR and GtrS form indeed a TCS. GtrS is a transmembrane sensor kinase that contains a periplasmic ligand binding domain. Efficient GtrS autophosphorylation and transphosphorylation to the GltR response regulator have been observed. GtrS recognizes specifically 2‐ketogluconate and 6‐phosphogluconate (Table 1), causing a modulation of its autokinase activity, leading in turn to changes in GltR transphosphorylation activity (Fig. 4).

GltR interacts with different promoters regulating the expression of the *oprB*,* glk*,* edd* and *gap‐1* genes (Fig. 3). Most interestingly, GltR also binds to the P_*toxA*_ promoter regulating *toxA* expression, underlining the interconnectivity of regulatory mechanisms for glucose metabolism and exotoxin A expression in *P. aeruginosa* (Daddaoua *et al*., 2014). GltR acts as a transcriptional repressor that is released from DNA upon phosphorylation and the consensus sequence for GltR was determined to be 5′‐tgGTTTTTc‐3′ (Table 1 and 2; Daddaoua *et al*., 2014).

**Figure 6:**
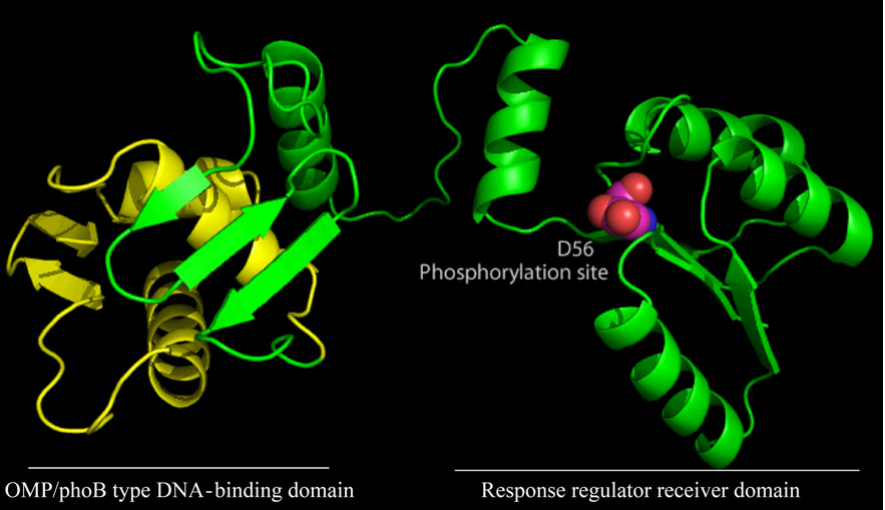
Homology model of the GltR regulator involved in the regulation of carbohydrate catabolism pathways and exotoxin A expression. The model has been generated using the I‐TASSER server software using the RegX3 from *Mycobacterium tuberculosis* structure as a template (PDB: 2OQR). The phosphoryl group accepting aspartate residue (D56) in the receiver domain is highlighted. The green part of the structure indicates the response regulator receiver domain, and the yellow part indicates the OMP/phoB type DNA‐binding domain.

The inspection of the homology model generated using OMPR/phoB type DNA‐binding domain (amino acids 134‐234) PDB: 2OQR) (King‐Scott *et al*., 2007) as template (Fig. 6) suggests that residues Ile‐200, Met‐203 and Arg‐211 are involved in recognition of its DNA‐binding site (Fig. 5).

This review summarizes the current knowledge on the specific regulatory circuits that govern glucose metabolism in Pseudomonads. However, there is also evidence for global regulation and interconnection with other processes, although the precise mechanisms have not been elucidated yet. Available information suggests the ketogluconate branch plays a role in the control virulence; and An and Moe (2016) showed that Gcd levels varied significantly with the carbon source used by *Pseudomonas*, showing that expression in glucose was higher than in glycerol, LB and citrate, which was consistent with the requirements in glucose dehydrogenase activity. These authors also showed that *gcd* expression was downregulated by inorganic phosphate, a demonstration of interconnection among metabolism of different nutrients.

## Concluding remarks

Electrophoresis mobility shift assay, footprinting, isothermal titration calorimetry, classical expression assays and computer modelling analysis allowed to gain insight into the molecular mechanisms that govern carbohydrate degradation pathways in *Pseudomonas*. These analyses have answered a number of questions that emerged from the early biochemical studies. This review has focussed in the latest advances in the regulatory mechanism rather than in the carbohydrate metabolism by itself. The complexity of the glucose degradation in *Pseudomonas* is given by the fine regulation of glucose fluxes among three different convergent pathways and how these pathways are coordinately expressed.

*Pseudomonas aeruginosa* is among the most feared human pathogens. Importantly, the transcriptional regulation of glucose metabolism in *P. aeruginosa* is intimately linked to bacterial virulence. So far, five specific regulatory systems have been shown to modulate glucose metabolism and transport (HexR, PtxS, PtxR, GtrS/GltR and GntR), of which two, PtxS and GtrS/GltR, were found to regulate, directly or through PtxR, the expression of *toxA,* encoding the primary virulence factor; exotoxin A. There is thus a functional link between glucose assimilation and exotoxin A production in *P. aeruginosa*. The physiological relevance of this connection remains unclear, and it needs to be established whether a similar relationship is also found in other bacteria.

In general, the effector molecules of most signal transduction systems are unknown. However, the effectors for four of the five systems have been established. In all cases, these effectors were intermediates of the glucose metabolism, whereas glucose itself is not recognized by any of the sensor proteins. 2KG and 6‐phosphogluconate play central roles as they are recognized by two different sensor proteins. In the case of 6‐phosphogluconate, this role may be related to the fact that all three glucose metabolism pathways converge in this metabolite. The importance of 2KG as an effector molecule may suggest that the corresponding metabolic route is of particular relevance. The structural basis for their recognition is different as, for example, 2KG is recognized by a periplasmic binding protein type of sensor domain (Pfam00532) at PtxS, whereas the GtrS sensor domain remains unannotated.

Another interesting feature is the cellular compartment at which the signals are of sensed. Whereas 2KG and 6‐phosphogluconate are sensed in the cytosol by PtxS and GntR, respectively, both ligands are sensed by GtrS which contains a periplasmic sensor domain. TCS represents a genetic and metabolic burden as compared to OCS, but its capacity to sense ligands in the extracytosolic space is considered as a major advantage over OCSs. In the present case, a system based on the dual sensing of the same effector in different cellular compartments has evolved, which may suggest that sampling information on intermediate concentrations in both cell compartments is advantageous.

Taken together, the molecular mechanism of transcriptional regulation of carbohydrate metabolism in *P. aeruginosa* and *P. putida* can be considered a model system to understand complex regulatory processes in bacteria.

## Conflict of interest

None declared.
